# Endogenous Interferon-β-Inducible Gene Expression and Interferon-β-Treatment Are Associated with Reduced T Cell Responses to Myelin Basic Protein in Multiple Sclerosis

**DOI:** 10.1371/journal.pone.0118830

**Published:** 2015-03-04

**Authors:** Lars Börnsen, Jeppe Romme Christensen, Rikke Ratzer, Chris Hedegaard, Helle B. Søndergaard, Martin Krakauer, Dan Hesse, Claus H. Nielsen, Per S. Sorensen, Finn Sellebjerg

**Affiliations:** 1 Danish Multiple Sclerosis Center, Department of Neurology, Rigshospitalet, University of Copenhagen, Copenhagen, Denmark; 2 Institute for Inflammation Research, Department of Rheumatology, Rigshospitalet, University of Copenhagen, Copenhagen, Denmark; University of Düsseldorf, GERMANY

## Abstract

Autoreactive CD4^+^ T-cells are considered to play a major role in the pathogenesis of multiple sclerosis. In experimental autoimmune encephalomyelitis, an animal model of multiple sclerosis, exogenous and endogenous type I interferons restrict disease severity. Recombinant interferon-β is used for treatment of multiple sclerosis, and some untreated multiple sclerosis patients have increased expression levels of type I interferon-inducible genes in immune cells. The role of endogenous type I interferons in multiple sclerosis is controversial: some studies found an association of high expression levels of interferon-β-inducible genes with an increased expression of interleukin-10 and a milder disease course in untreated multiple sclerosis patients, whereas other studies reported an association with a poor response to treatment with interferon-β. In the present study, we found that untreated multiple sclerosis patients with an increased expression of interferon-β-inducible genes in peripheral blood mononuclear cells and interferon-β-treated multiple sclerosis patients had decreased CD4^+^ T-cell reactivity to the autoantigen myelin basic protein *ex vivo*. Interferon-β-treated multiple sclerosis patients had increased *IL10* and *IL27* gene expression levels in monocytes *in vivo*. *In vitro*, neutralization of interleukin-10 and monocyte depletion increased CD4^+^ T-cell reactivity to myelin basic protein while interleukin-10, in the presence or absence of monocytes, inhibited CD4^+^ T-cell reactivity to myelin basic protein. Our findings suggest that spontaneous expression of interferon-β-inducible genes in peripheral blood mononuclear cells from untreated multiple sclerosis patients and treatment with interferon-β are associated with reduced myelin basic protein-induced T-cell responses. Reduced myelin basic protein-induced CD4^+^ T-cell autoreactivity in interferon-β-treated multiple sclerosis patients may be mediated by monocyte-derived interleukin-10.

## Introduction

Multiple sclerosis (MS) is a disease in which inflammatory focal lesions cause demyelination, axonal loss and glial scars in the central nervous system leading to chronic disability of the patient. The etiology of MS is unknown. It is believed, however, that systemic activation of myelin-reactive T- helper (T_H_)1 and T_H_17 cells and their reactivation within the CNS is crucial for induction of disease activity [1–3].

Type I interferons (IFN) are important cytokines in innate and antiviral immune responses, but they also have a role in adaptive immunity. Engaging their common cell surface receptor, type I IFNs modulate the biological activity of immune cells by induction and repression of myriads of genes [4]. One type I IFN, IFN-β, is a commonly used first line treatment in relapsing-remitting MS (RRMS), and reduces the number of relapses and inflammatory brain lesions [5,6]. Recent studies suggested that type I IFNs may modulate the secretion of cytokines by antigen-presenting cells (APCs) to interfere with T-cell activation [7–10]. In an animal model of MS, experimental autoimmune encephalomyelitis (EAE), exogenous and endogenous type I IFNs restrict disease severity [7,11–14], possibly mediated by monocytes [7,12]. However, studies disagree on whether or not type I IFNs influence T-cell activation in EAE. Furthermore, a recent study indicated that the effect of type I IFNs may differ in EAE induced by T_H_1 or T_H_17 cells, attenuating T_H_1-mediated while increasing severity in T_H_17-mediated EAE [15].

Two independent studies found spontaneous expression of IFN-β-inducible genes in peripheral blood mononuclear cells (PBMC) from subgroups of untreated MS patients [16,17]. Since then, high expression levels of IFN-β-inducible genes have been associated with disease control and increased expression of genes encoding immunoregulatory cytokines, including interleukin (IL)-10, in untreated RRMS [18–20]. Accordingly, we found that the expression of *IL10* was decreased in patients who had developed neutralizing anti-IFN-β antibodies following treatment with IFN-β [18]. The effect of endogenous type I IFNs on T-cell activation in MS is unknown.

The present study evolved from our initial finding that CD4^+^ T-cell activity to myelin basic protein (MBP) in untreated MS patients *ex vivo* was associated with low endogenous expression of IFN-β-inducible molecules in PBMCs. First, we confirmed that type I IFNs may interfere with CD4^+^ T-cell reactivity to MBP in IFN-β treated MS. Second, we assessed the effects of IFN-β treatment on the *in vivo* mRNA expression of cytokines and transcription factors involved in T-cell activation in whole blood and in the major blood cell subtypes. Finally, we showed that immunoregulatory cytokines, which were strongly induced in monocytes in IFN-β-treated MS, interfered with the activation of antigen-specific CD4^+^ T-cells *ex vivo*, and demonstrated that monocytes had an immunoregulatory effect on MBP-reactive T cells.

## Methods

### Ethics statement

The study was approved by the regional scientific Ethics Committee of Copenhagen County (protocol KF 01–0141/95 and KF 01–314009) and all persons participating in this study provided written informed consent.

### Patients

The different sub-studies of the presented work were conducted on different patient populations (Table 1): sub-study 1) We determined gene expression levels and identified differentially expressed genes in 4 patients with T cells responding to MBP and 13 MBP non-responders by analyzing the results from previous studies on MBP-induced T-cell reactivity [21] and corresponding, available DNA array studies of gene expression in peripheral blood mononuclear cells (PBMCs) in these untreated MS-patients (Table 1) [18]. To identify IFN-β induced genes in MBP responders and MBP non-responders from the sub-study 1 population in the present work, we used the results from Affymetrix studies on 23 IFN-β treated patients as reference (data and patient characteristics are given elsewhere) [22]; sub-study 2) Treatment effects of IFN-β on antigen-induced T cell proliferation *ex vivo*, intracellular cytokine expression in antigen-specific T-cells *ex vivo* or gene expression in CD4^+^ T-cells or monocytes were analyzed in randomly obtained, unselected blood samples from a group of 24 IFN-β treated and 18 untreated RRMS patients (Table 1); sub-study 3) mRNA expression levels in randomly obtained, unselected whole blood samples were measured in two statistically independent groups: in the discovery group samples were obtained from 26 IFN-β-treated and 25 untreated RRMS patients, and in the validation group samples were obtained from 14 RRMS patients before and later than 6 months after initiation of IFN-β treatment (Table 1); sub-study 4) we compared mRNA-expression levels in whole blood, PBMCs, CD4^+^ and CD8^+^ T-cells, NK-cells, B-cells, dendritic cells and monocytes using blood samples from 4 untreated and 4 IFN-β-treated patients (Table 1); sub-study 5) for functional cell studies *in vitro* we used blood samples from 11 individuals (compound data from 6 healthy volunteers, 4 untreated MS patients and 1 IFN-β-treated MS patient) and different conditions were tested in at least four independent experiments. RRMS patients had not had a relapse and had not received treatment with glucocorticoids within a 3 months period prior to sampling.

**Table 1:** Characteristics of relapsing-remitting multiple sclerosis (RRMS) patients included in this study.

		Substudy-1 population		Substudy-2 population		Substudy-3 population			Substudy-4 population	
		Untreated RRMS				Discovery group		Validation group		
		MBP responders	MBP non-responders	Untreated RRMS	IFN-β treated RRMS	Untreated RRMS	IFN-β treated RRMS	RRMS pre- and on IFN-β treatment	Untreated RRMS	IFN-β treated RRMS
**Patient characteristics**	N	4	13	18	24	25	26	14	4	4
	Age (years)	29 (26–36)	37 (32–42)	34 (27–41)	36 (32–40)	36 (29–41)	35 (32–44)	29 (26–37)	NA	NA
	Female/male	3/1	8/5	14/4	18/6	18/8	16/9	8/6	NA	NA
	EDSS	0.5 (0–2)	1.0 (0.5–3)	1.5 (1.3–2.8)	1.5 (1.5–2.5)	2.0 (1.0–3.5)	2.5 (1.5–4)	1.0 (1.0–3.0)	NA	NA
	Disease duration (years)	6.5 (2–11)	4 (2.5–6.5)	2.5 (1–8)	7 (4–10)	5 (4–9)	6 (5–10)	2(2–6)	NA	NA
**Substudies**	Differentially expressed genes in DNA-array studies	n = 4	n = 13	ND	ND	ND	ND	ND	ND	ND
	T-cell proliferation (MBP)	ND	ND	n = 13	n = 18	ND	ND	ND	ND	ND
	T-cell proliferation (TT)	ND	ND	N = 12	N = 18	ND	ND	ND	ND	ND
	Intracellular cytokine T-cell assay	ND	ND	n = 11	n = 22	ND	ND	ND	ND	ND
	mRNA expression studies on CD4^+^ T-cells	ND	ND	n = 11	n = 12	ND	ND	ND	ND	ND
	mRNA expression studies on monocytes	ND	ND	n = 11	n = 14	ND	ND	ND	ND	ND
	mRNA-expression in whole blood using PCR	ND	ND	ND	ND	n = 25	n = 26	n = 14	ND	ND
	mRNA-expression in whole blood and cell-subsets	ND	ND	ND	ND	ND	ND	ND	n = 4	n = 4

Values are given as median with interquartile range in parenthesis. RRMS = relapsing remitting multiple sclerosis; MBP = myelin basic protein; TT = tetanus toxoid; EDSS = Expanded Disability Status Scale; IFN-β = interferon-β; ND = not done; NA = not applicable.

### Blood samples and sample preparation

Blood samples were obtained from IFN-β-treated patients 36 to 48 hours after their last injection of IFN-β. For whole blood gene expression analysis, blood was collected in PAXgene tubes (PreAnalytiX, Germany). For all other purposes, blood samples were drawn in BD Vacutainer EDTA tubes (BD Biosciences, Denmark). PBMCs were promptly separated by density gradient centrifugation, washed twice in cold PBS, passed through a MACS Pre-Separation Filter (Miltenyi Biotec, Germany) and stored at 4°C for further processing the same day.

### Separation of blood cell-subsets

CD4^+^ and CD8^+^ T-cells, NK-, B- and dendritic cells and monocytes were enriched from 50–100 x 10^6^ PBMCs. Freshly isolated PBMCs were incubated with immunomagnetic beads—according to the manufacturer’s instructions—using CD4^+^ T Cells Isolation Kit II, CD8^+^ T Cells Isolation Kit II, CD19 MicroBeads, NK Cell Isolation Kit, Blood Dendritic Cell Isolation Kit II and CD14 MicroBeads (all from Miltenyi Biotec). Labeled PBMCs were applied to an AutoMACS cell-separator (Miltenyi Biotec) to isolate 2 x10^5^ to 1 x 10^6^ target cells.

### Gene expression analysis

Total RNA from blood cell subsets and PBMCs was extracted with the Pico Pure RNA Isolation Kit (Arcturus, USA), RNA from PAXgene tubes was extracted with the PAXgene RNA Blood kit (PreAnalytiX) and stored at -80°C. For gene expression analysis of whole blood and explorative cell-sorting experiments, cDNA was synthesized with High Capacity cDNA RT kit (Applied Biosystems, USA); for gene expression analysis of CD4^+^ T-cells and monocytes, cDNA was synthesized with qScript cDNA SuperMix (Quanta BioSciences, USA). Quantitative real-time PCR (qPCR) was run on an ABI 7500 Real Time PCR System (Applied Biosystems) and predesigned and validated TaqMan technology primers and hydrolysis probes were used (S1 Table; Applied Biosystems). Gene expression in each sample of the target mRNA, relative to an endogenous reference gene (ΔC_t-sample_), was compared to a calibrator consisting of pooled cDNA made of PBMC from healthy volunteers (ΔC_t-pool_). For gene expression analysis of whole blood and explorative cell sorting experiments we used *GAPDH* as reference genes, for gene expression analysis of CD4^+^ T-cells and monocytes we used *CASC3* and *UBE2D2* as reference genes. Gene expression levels are given as normalization ratio (NR) calculated by: NR = 2^-ΔCt(sample) - ΔCt(pool)^ [23]. Gene expression in PBMCs was analyzed on the Affymetrix Human Genome Focus Gene Chip as previously described [24].

### Cell culture

Carboxyfluorescein diacetate succinimidyl (CFSE; Molecular Probes, Invitrogen, Denmark) was added to a final concentration of 1μM to freshly isolated PBMCs in PBS. After 2.5 minutes of incubation at room temperature (RT), cells were washed in culture medium (CM; RPMI1640-Glutamax (Invitrogen, Denmark) supplemented with 5% (v/v) human serum albumin (HSA; Sigma, USA) and penicillin (50 units/ml) and streptomycin (50 μg/ml; both Invitrogen, Denmark)). Finally, the cells were resuspended in CM and transferred to flat bottom culture plates (Cellstar; Greiner bio-one, Germany): 5x10^5^ PBMCs in 200 μl CM per well on a 96-well plate or 1.7x10^6^ PBMCs in 714 μl CM per well on 48-well plates. For antigen-specific activation, tetanus toxoid (TT: 10 μg/ml; Statens Serum Institute, Denmark) or myelin basic protein (MBP: 30 μg/ml; HyTest, Finland) was added to the CM. For some studies we added IL-10 (5 ng/ml), IL-27 (40 ng/ml), goat-anti-IL-10 (6 μg/ml), goat-anti-IL-27 (6 μg/ml) or goat-IgG1(6 μg/ml) (all from R&D-Systems, UK) to the CM. Monocyte-depleted PBMCs were obtained using CD14 MicroBeads, MS-Column and a MiniMACS Separation Unit (Miltenyi-Biotec). Cell cultures were incubated 7 days at 37°C in a humidified 5% CO_2_ atmosphere. For intracellular cytokine staining at day 7, 100 μl of the supernatant was replaced with fresh CM containing 10 ng/ml phorbol 12-myristate 13-acetate (PMA; Sigma) and ionomycin 1mM (Sigma). After 1 hour, brefeldin A (Sigma) was added to a final concentration of 5 μg/ml for another 4 hours.

### Flow cytometry analysis of CD4^+^ T-cell reactivity to MBP and TT

Cells were harvested, washed twice in PBS at 4°C and stained for CD3, CD4, CD8 and CD19 with fluorochrome-conjugated antibodies and for vitality with a Live/Dead staining dye (S2 Table) for 30 minutes in the dark at 4°C. The cells were subsequently washed in FACS-PBS (PBS/ 1% (w/v) HSA/2nM EDTA (FACS-PBS)) and resuspended in FACS-PBS for immediate flow-cytometric analysis. For flow cytometry we used a BD FACSCanto II and the BD FACSDiva Software 6.1.2 (all from BD Biosciences, Denmark). Proliferating CD4^+^ T-cells were assessed as percentage of CFSE-diluted cells within the CD3^+^CD4^+^ cell population. In some *in vitro* studies proliferating CD4^+^ T-cells were assessed as the percentage of CFSE-diluted cells within the CD3^+^CD8^-^ cell population.

To assess the intracellular cytokine production in proliferating CD4^+^ T-cells, the cells were surface stained for vitality with Live/Dead staining dye and for CD3, CD8 and CD19 with fluorochrome conjugated antibodies (S1 Table). Thereafter, the cells were fixed and permeabilized with FOXP3 permeabilization kit (BioLegend, USA) and stained for 30 minutes at RT with antibody combinations of: anti-17A-PE and anti-IFN-γ APC; anti-IL-4-PE and anti-TNF-α-APC; anti-IL-10-PE and anti-IL13-APC; anti-FoxP3 Alexa-Fluor 647 (S1 Table). As control for non-specific background- fluorescence and non-specific antibody binding, tetanus-toxoid (TT)-stimulated cells were stained with isotype matched control antibodies.

### Data analysis and statistical methods

Data are given as median with inter-quartile range (IQR) or presented as boxplots: boxes represent interquartile range, median value indicated as a line, whiskers represent range, ^₀^ = outliers, * = extremes. Group differences were analyzed by non-parametric tests: Mann-Whitney U test for unpaired samples; Wilcoxon Signed Ranks test for paired samples. Data on cell culture were analyzed by paired and unpaired, two-tailed t-tests due to the limited number of experiments for some setups. When observations (unpaired analysis) and differences (paired analysis) had unequal variances or were not normally distributed, data were transformed by taking the root or cubic root prior to statistical testing. P-values below 0.05 were considered significant. Data were processed using Microsoft Excel and PASW 18.0.1.

## Results

### Low expression levels of IFN-β-inducible genes are associated with increased MBP-induced CD4^+^ T-cell proliferation *ex vivo*

Out of 17 untreated RRMS patients included in a previous study, 4 patients responded to myelin basic protein (MBP) with CD4^+^ T-cell proliferation (sub-study 1 population)[21]. Comparing PBMC gene expression in the 4 MBP responders with gene expression in the 13 MBP non-responders [18], we found that out of 3284 genes, calling as present in all patients according to Affymetrix algorithms, 75 genes were differentially expressed in the two groups (Table 2a; p<0.01 and expressed at 1.5-fold higher levels in one of the groups (highlighted with bold letters); microarray data are available in the ArrayExpress database (www.ebi.ac.uk/arrayexpress) under accession number E-MTAB-2707).

**Table 2:** Association between endogenous expression of IFN-β-inducible genes in peripheral blood mononuclear cells with T-cell reactivity to myelin basic protein (MBP) and tetanus toxoid (TT) in untreated MS patients.

		Gene expression ratio (MBP responders/ non-responders)^a^		IFN-ß treatment induced changes in gene expression^b^		Gene expression ratio (TT proliferation high/ low)^c^	
Representative public ID	Gene symbol	Expression ratio	P-value	Gene expression	FDR	Gene expression	P-value
AA393940	---	**2,38983**	**0,00004**	1,05436	0,71439	0,65885	0,07972
AF127481	---	**0,66090**	**0,00568**	0,80634	0,23709	1,72837	0,09079
AL527773	ABR	**0,60767**	**0,00796**	0,94413	0,44159	0,79811	0,44018
S69189	ACOX1	**0,60974**	**0,00695**	0,85280	0,13347	0,73019	0,19905
NM_006395	ATG7	**0,53188**	**0,00587**	1,20249	0,00433	0,71277	0,30945
U72937	ATRX	**0,65347**	**0,00415**	0,72367	0,00094	0,65718	0,12609
AB035482	C1orf38	**0,65053**	**0,00801**	1,63014	0,00000	0,80314	0,38388
U62027	C3AR1	**0,59222**	**0,00348**	2,64914	0,00001	0,68092	0,19756
U47924	CD4	**0,62806**	**0,00558**	0,92128	0,56399	0,74489	0,22181
NM_001784	CD97	**0,64874**	**0,00816**	1,35297	0,00026	0,78190	0,32496
AL564683	CEBPB	**0,63381**	**0,00476**	1,50631	0,00014	0,77715	0,08505
NM_005195	CEBPD	**0,63337**	**0,00417**	1,61098	0,00140	0,77148	0,30036
NM_006090	CEPT1	**0,51559**	**0,00032**	1,05430	0,64946	0,67176	0,16979
NM_000760	CSF3R	**0,56506**	**0,00785**	1,56528	0,00008	0,76494	0,36178
NM_004383	CSK	**0,50162**	**0,00561**	1,12576	0,18584	0,55615	0,14777
N25915	CUGBP1	**0,49958**	**0,00484**	0,74453	0,03732	0,55201	0,15624
NM_004417	DUSP1	**0,44522**	**0,00398**	1,52685	0,00020	0,56529	0,11542
NM_001953	ECGF1	**0,51953**	**0,00732**	4,53205	0,00002	1,12746	0,76894
BC000533	EIF3S8	**0,63224**	**0,00664**	0,79373	0,01835	0,74438	0,35954
NM_012177	FBXO5	**0,66612**	**0,00629**	0,85859	0,27780	**0,58712**	**0,00558**
AW001443	GGA1	**0,49321**	**0,00502**	0,70943	0,00408	0,64771	0,26468
AW009695	GGA1	**0,48944**	**0,00551**	0,70032	0,00746	0,60170	0,24124
NM_004126	GNG11	**1,53490**	**0,00020**	0,81936	0,01488	1,25208	0,25286
BC000324	GRN	**0,60910**	**0,00900**	2,15801	0,00000	0,96352	0,88274
NM_022460	HS1BP3	**0,61000**	**0,00873**	1,09055	0,26549	0,78109	0,25038
AF141870	ILF3	**0,46941**	**0,00869**	0,73496	0,03630	0,52984	0,16518
NM_002199	IRF2	**0,61305**	**0,00405**	2,13032	0,00042	0,62974	0,12245
NM_006084	ISGF3G	**0,65931**	**0,00629**	1,48177	0,00002	0,81538	0,35916
NM_002229	JUNB	**0,42768**	**0,00074**	2,00080	0,00000	0,54768	0,06116
NM_005354	JUND	**0,58269**	**0,00860**	1,34615	0,01096	0,63146	0,21424
NM_007054	KIF3A	**0,62358**	**0,00228**	0,62132	0,00137	0,64832	0,01398
NM_016285	KLF12	**0,60366**	**0,00182**	0,67407	0,00478	0,63583	0,06954
BF514079	KLF4	**0,59463**	**0,00091**	1,32022	0,00059	0,71383	0,12362
NM_005461	MAFB	**0,60432**	**0,00356**	3,03565	0,00000	0,72817	0,20138
NM_002406	MGAT1	**0,55525**	**0,00223**	1,45918	0,00004	0,70792	0,19953
NM_005931	MICB	**0,62026**	**0,00773**	1,05054	0,69661	0,62686	0,12993
NM_014381	MLH3	**1,55617**	**0,00037**	0,98685	0,89715	1,30512	0,22600
NM_012331	MSRA	**0,66622**	**0,00478**	0,95432	0,51950	0,74943	0,08485
NM_017458	MVP	**0,55557**	**0,00277**	1,89293	0,00000	0,73133	0,26680
U70451	MYD88	**0,57903**	**0,00055**	1,91913	0,00000	0,73916	0,17010
NM_002505	NFYA	**0,49007**	**0,00928**	0,92316	0,55970	0,48639	0,13324
NM_013392	NRBP1	**0,52913**	**0,00542**	1,05099	0,58002	0,70485	0,30052
AI824012	NRIP1	**0,53985**	**0,00219**	1,67004	0,00233	0,60243	0,08224
NM_002534	OAS1	**0,40953**	**0,00229**	6,83506	0,00000	0,62769	0,25810
NM_016817	OAS2	**0,54858**	**0,00293**	4,85365	0,00000	0,77768	0,43984
NM_006187	OAS3	**0,56192**	**0,00701**	7,09851	0,00000	0,79581	0,45168
AF242521	OAZ2	**0,62811**	**0,00292**	1,11296	0,05416	0,78569	0,35423
NM_023914	P2RY13	**0,53025**	**0,00107**	1,61084	0,00057	0,74296	0,25271
NM_002656	PLAGL1	0,71437	0,01698	1,23990	0,07508	**0,59712**	**0,00765**
NM_018444	PPM2C	**0,55737**	**0,00865**	0,89289	0,33921	0,67981	0,24250
NM_007318	PSEN1	**0,61678**	**0,00925**	1,05178	0,46844	0,66741	0,15644
NM_002806	PSMC6	0,79988	0,16321	0,79397	0,00226	**0,66459**	**0,00427**
NM_002872	RAC2	**0,66609**	**0,00465**	0,96417	0,69396	0,68330	0,08692
NM_002902	RCN2	0,81450	0,11315	0,50180	0,00001	**0,60390**	**0,00465**
AI263909	RHOB	**0,37747**	**0,00029**	3,49399	0,00001	0,55096	0,10617
NM_012249	RHOQ	**0,59453**	**0,00037**	1,13092	0,19030	0,71506	0,14372
BE897886	RHOQ	**0,54734**	**0,00163**	1,06784	0,42048	0,56116	0,07599
NM_002939	RNH1	**0,52587**	**0,00221**	1,44191	0,00004	0,92825	0,81932
NM_000979	RPL18	**1,50894**	**0,00011**	0,69713	0,00006	1,33955	0,02902
NM_003942	RPS6KA4	**0,58647**	**0,00545**	1,30240	0,05970	0,71857	0,34645
AI871457	SFRS8	0,77329	0,02205	0,74400	0,00536	**0,64223**	**0,00582**
NM_005627	SGK	**0,56251**	**0,00198**	0,67001	0,00243	0,73625	0,28065
NM_020979	SH2B2	**0,62734**	**0,00053**	1,65552	0,00000	0,80701	0,28688
AI025519	SLC26A2	**0,58834**	**0,00438**	0,62356	0,00097	0,62053	0,13411
AA524345	SNX4	**0,65104**	**0,00840**	0,87639	0,20487	0,61664	0,07937
X15132	SOD2	0,67714	0,03673	1,60177	0,00032	**0,56143**	**0,00801**
AL442077	SPFH2	**0,62805**	**0,00197**	0,88592	0,12560	0,69761	0,07905
M97935	STAT1	**0,54867**	**0,00826**	2,23157	0,00000	0,69476	0,32416
NM_005131	THOC1	**0,64186**	**0,00742**	0,56570	0,00006	0,61169	0,06322
NM_003266	TLR4	**0,61293**	**0,00465**	1,45846	0,00045	0,68766	0,14945
NM_016610	TLR8	**0,51446**	**0,00730**	1,18074	0,08572	0,63275	0,25378
NM_001243	TNFRSF8	**0,59619**	**0,00229**	0,94210	0,62315	0,70852	0,17841
NM_025195	TRIB1	**0,57316**	**0,00247**	1,77640	0,00045	0,61108	0,07589
AI363270	TRIM38	**0,61895**	**0,00611**	1,45895	0,00016	0,65750	0,16616
NM_030912	TRIM8	**0,64113**	**0,00047**	1,11388	0,23857	0,76183	0,12478
NM_007124	UTRN	**0,55447**	**0,00563**	1,07708	0,47925	0,57623	0,09694
NM_004184	WARS	**0,55612**	**0,00991**	3,98419	0,00000	0,92506	0,81254
NM_014795	ZFHX1B	**0,56386**	**0,00468**	1,16480	0,31749	0,52589	0,06223
NM_003407	ZFP36	**0,54741**	**0,00230**	1,42917	0,00657	0,59914	0,09480
BG250310	ZFP36L1	**0,62283**	**0,00140**	1,05199	0,61205	0,65364	0,10535

(a) In a previous study, 4 out of 17 patients responded to MBP with CD4^+^ T-cell proliferation ex vivo [21]. By DNA array analysis of gene expression [18], we found that 75 out of 3284 genes were more than 1.5-fold differentially expressed (p<0.01) in the 4 patients who had CD4^+^ T-cell reactivity to MBP as compared to the 13 patients who did not show CD4^+^ T-cell reactivity to MBP (differentially expressed genes are highlighted with bold letters). Data were obtained from the substudy-1 population and are given as ratio of the mean gene expression in both groups.

(b) We compared these results with data on IFN-β treatment-induced gene-expression levels in 23 patients from a recent study from our group [22]: 23 out of the 75 (31%) genes differentially expressed in patients with MBP-induced T-cell proliferation were induced at least 1.5-fold in patients treated with IFN-β (false discovery rate< 1% for all genes). Out of the remaining 3209 genes 171 (5.3%) were similarly induced after treatment with IFN-β (X^2^ = 84.64, df = 1, p<0.001).

(c) Data on TT reactivity were available from 12 patients from the sub-study 1 population [21]. Dividing the patients into two groups according to the level of TT reactivity (6 TT high responders vs. 6 TT low responders), we calculated the gene-expression ratio and identified 6 genes that were more than 1.5-fold differentially expressed (p<0.01; bold letters). (b) 4 out of these 6 genes were also significantly regulated by treatment with IFN-β, although only one of these genes was induced more than 1.5-fold.

Many of the genes expressed at lower levels in patients with MBP-induced T cell proliferation were IFN-β-inducible genes, e.g., *OAS1*, *OAS2*, *OAS3* and *STAT1*. We therefore compared these results from the sub-study 1 population with data on IFN-β-treatment-induced gene expression levels in 23 patients from a recent study from our group [22]. Hereby we found that 23 out of the 75 genes (31%), differentially expressed in patients with MBP-induced T-cell proliferation, were induced at least 1.5-fold in patients treated with IFN-β (false discovery rate< 1% for all genes; Table 2b). In contrast, only 171 of the remaining 3209 genes (5.3%) were similarly induced after treatment with IFN-β (X^2^ = 84.64, df = 1, p<0.001). Since the 23 differentially expressed genes were all expressed at lower levels in patients with MBP-induced T-cell proliferation, these results suggest an association between low endogenous type I IFN activity and T-cell reactivity to MBP in untreated MS patients. Data on TT-induced T-cell reactivity were available from 12 patients from the sub-study 1 population. Dividing the patients into two groups according to the level of TT-induced T-cell proliferation (6 TT-high responders vs. 6 TT-low responders), we identified 6 genes that were differentially expressed (Table 2c; p<0.01 and expressed at 1.5-fold higher levels in one of the groups). We found that 4 out of these 6 genes were also significantly regulated by treatment with IFN-β (false discovery rate< 1%), although only one of these genes was induced more than 1.5-fold (Table 2b).

### IFN-β treated patients have decreased MBP-induced proliferation of CD4^+^ T-cells *ex vivo*

**Fig 1:**
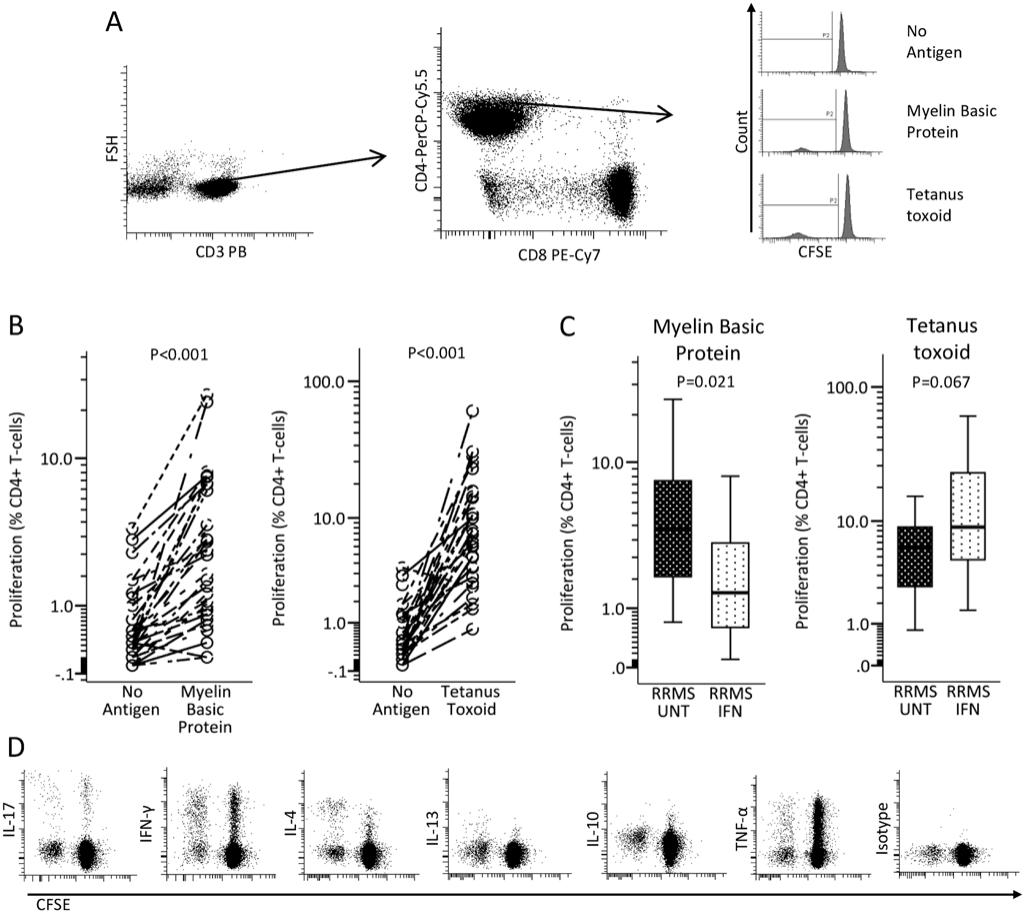
Responsiveness of CD4^+^ T-cells to myelin basic protein (MBP) and tetanus toxoid (TT) *ex vivo*. (A) Representative dot plots and histograms showing the gating strategy for measurement of CD4^+^ T-cell proliferation—before T-cells were identified debris, dead cells and CD19^+^ cells had been excluded. (B) Graphs shows significant proliferative response of CD4^+^ T-cell to TT (n = 30; pooled data from 12 untreated and 18 interferon (IFN)-β treated relapsing remitting multiple sclerosis (RRMS) patients from the sub-study 2 population) and MBP (n = 31;pooled data from 13 untreated and 18 IFN-β treated RRMS patients from the sub-study 2 population). (C) Boxplots compare proliferative CD4^+^ T-cell responses to MBP in 13 untreated and 18 IFN-β treated patients and to TT in 12 untreated and 18 IFN-β-treated MS patients (sub-study 2 population). Boxes represent interquartile range, median value indicated as a line; whiskers represent range. (D) Representative plots of flow cytometry data to assess intracellular-cytokine production in proliferating CD3^+^CD8^-^cells (data given in Table 3). Statistical analysis was by (B) paired and (C) unpaired t-test.

We hypothesized that if increased expression of IFN-β-inducible genes in untreated MS patients interfered with CD4^+^ T-cell reactivity to MBP *ex vivo*, IFN-β treatment might also do so. We modified our PBMC based CFSE-assay; CFSE-labeling was optimized to diminish toxic effects of CFSE in labeled cells [25] and the amount of serum in culture media was reduced to 5%. Proliferation was assessed as percent CD3^+^CD4^+^ cells with diluted CFSE staining (Fig. 1A). These studies were made on patients from the sub-study 2 population. In most individuals, we were able to detect proliferation of CD4^+^ T-cells after 7 days in response to MBP (n = 31) and tetanus toxoid (TT; n = 30; Fig. 1B). Compared to 13 untreated MS patients, 18 IFN-β treated patients had a significantly lower proliferative response of CD4^+^ T cells to the self-antigen MBP, while the proliferative response to the recall antigen TT was not significantly different in 12 untreated MS patients as compared to 18 IFN-β treated patients (Fig. 1C).

**Table 3:** Phenotype of tetanus toxoid (TT)- and myelin basic protein (MBP)-reactive CD3^**+**^CD8^-^ T-cells, from untreated (UNT) and interferon (IFN)-β-treated patients with relapsing-remitting multiple sclerosis (RRMS).

	Tetanus Toxoid		Myelin Basic Protein	
	RRMS UNT (n = 11)	RRMS IFN (n = 22)	RRMS UNT (n = 11)	RRMS IFN (n = 22)
	% proliferating CD8^-^ T-cells			
IL-17^+^	10.4 (2.3)	8.4 (1.4)	14.8 (4.5)	12.3 (1.6)
IFN-γ^+^	47.3 (7.3)	36.8 (3.4)	27.6 (5.4)	27.7 (3.5)
IFN-γ^+^IL-17^+^	0.6 (0.3)	0.3 (0.1)	0.1 (0.1)	0.1 (0.1)
IL-4^+^	17.1 (2.0)	13.7 (1.8)	13.5 (2.4)	16.9 (2.1)
TNF-α^+^	48.9 (5.1)	35.3 (2.9)	38.2 (4.1)	30.7 (3.4)
IL-13^+^	22.8 (3.2)	14.9 (2.5)	14.0 (3.6)	7.2 (1.5)
IL-10^+^	8.6 (3.6)	4.8 (1.2)	6.0 (1.7)	7.2 (1.4)

CFSE-labeled PBMCs were incubated with TT and MBP for 7 days, restimulated with PMA/ ionomycin and brefeldin for 5 hours, permeabilized and stained for surface markers and intracellular cytokines with fluorochrome-conjugated antibodies. The percentage of interleukin (IL)-17A, IFN-γ, IL-4, tumor necrosis factor (TNF)-α, IL-13 and IL-10 expressing cells within CFSE-diluted (proliferating) CD3^+^CD8^-^ T-cell population was assessed by flow cytometry. Data were obtained from the sub-study 2 population and are given as mean (± standard error of the mean); there were no significant differences by unpaired t-test.

### Intracellular expression of T_H_1, T_H_2, T_H_17 and T_REG_ markers in MBP- and TT-responsive CD3^+^CD8^-^ T-cells from untreated and IFN-b treated MS patients *ex vivo*

As endogenous expression of IFN-β-inducible genes and IFN-β treatment appeared to affect the proliferative responsiveness of myelin-reactive CD4^+^ T-cells, we examined whether IFN-β treatment modified the phenotype of CD4^+^ T-cells proliferating in response to MBP and TT. On day 7, the antigen-challenged PBMCs were stimulated with PMA and ionomycin, permeabilized and stained for: IFN-γ (T_H_1); IL17A (T_H_17)); IL4 and IL13 (T_H_2); and the more broadly expressed cytokines IL-10 and TNF-α. As PMA strongly reduced the expression of CD4^+^ on T-cells, cytokine-production of CD4^+^ T-cells was assessed as cytokine-production in CD3^+^CD8^-^ T-cells (Fig. 1D). The cytokine expression pattern did not, however, differ between 11 untreated and 22 IFN-β-treated MS patients from the sub-study 2 population (Table 3).

### IFN-β treatment effects on T-cell signature molecules in whole blood

Next we wanted to examine how treatment with IFN-β may interfere with myelin-reactive T-cell activation. Thus, we systematically explored IFN-β treatment induced effects on the *in vivo* expression of transcription factors and cytokines, involved in T-cell activation and differentiation. We screened whole blood samples for IFN-β treatment effects in two independent cohorts (discovery group and validation group of the sub-study 3 population) by quantitative PCR (qPCR); only unidirectional changes in the gene expression with a p-value below 0.05 in both cohorts were considered to be significant (Table 4).

**Table 4:** Whole blood expression of genes associated with regulation, differentiation and activity of T-helper (T_**H**_) and T-regulatory cells in untreated (UNT) and interferon (IFN)-β-treated patients with relapsing remitting multiple sclerosis (RRMS).

		Discovery group			Validation group			
	Gen targets	Untreated RRMS (n = 25)	IFN-β-treatedRRMS (n = 26)	p-value	RRMS pre-treatment (n = 14)	IFN-β-treated RRMS (n = 14)	p-value	Pooled datap-value
**T**_**H**_ **1**	*IL12A*	1.3 (1.1–2.0)	0.8 (0.5–1.1)	p<0.001	1.6 (1.3–3.1)	1.0 (0.6–1.3)	NS	ND
	*TBX21*	1.0 (0.8–2.1)	0.6 (0.4–0.8)	p<0.001	1.4 (0.9–2.1)	1.1 (0.7–1.7)	NS	ND
	*HLX1*	1.3 (1.1–1.6)	0.7 (0.5–0.9)	p<0.001	1.4 (1.2–1.8)	0.9 (0.7–1.2)	P = 0.002	p<0.001
	*IL18*	1.4 (1.2–2.0)	1.1 (0.9–1.4)	NS	1.2 (0.9–1.5)	1.4 (1.0–1.5)	NS	ND
	*IFNG*	1.9 (1.4–2.9)	0.6 (0.4–1.3)	p<0.001	2.0 (1.6–3.2)	1.3 (0.5–1.8)	P = 0.003	p<0.001
**T**_**H**_ **17**	*IL1B*	1.1 (1.0–1.9)	0.8 (0.6–1.1)	P = 0.001	1.9 (1.3–2.8)	0.9 (0.6–1.3)	P = 0.002	p<0.001
	*IL23*	1.4 (1.2–2.2)	1.0 (0.8–1.2)	p<0.001	1.3 (0.8–2.1)	0.9 (0.7–1.0)	P = 0.003	p<0.001
	*IL1RN*	0.9 (0.7–1.1)	1.8 (1.2–2.2)	p<0.001	1.3 (0.7–2.6)	2.5 (2.2–3.3)	NS	ND
	*RORA*	1.0 (0.8–1.1)	0.8 (0.6–1.0)	P = 0.032	0.9 (0.5–1.1)	1.1 (0.7–1.2)	P = 0.041	ND
	*RORC*	1.4 (1.0–1.7)	0.9 (0.5–1.1)	p<0.001	1.4 (0.8–1.9)	1.0 (0.8–1.3)	NS	ND
**T**_**H**_ **2**	*GATA3*	1.5 (1.1–2.0)	1.3 (1.1–1.7)	NS	1.5 (1.1–1.7)	1.5 (1.0–1.7)	NS	ND
	*MAF*	2.3 (1.8–2.5)	1.4 (1.1–1.9)	p<0.001	1.9 (1.3–2.5)	1.5 (1.3–1.9)	NS	ND
**Others**	*EBI3*	1.5 (1.1–2.2)	0.9 (0.6–1.4)	P = 0.027	1.8 (0.9–5.2)	1.4 (0.7–2.6)	NS	ND
	*IL10*	1.5 (1.0–2.1)	3.0 (2.3–4.1)	p<0.001	2.4 (1.8–4.3)	4.5 (2.4–5.9)	P = 0.022	p<0.001
	*IL27*	1.7 (1.2–2.3)	4.0 (2.6–5.2)	p<0.001	1.9 (0.9–5.1)	4.5 (3.6–5.7)	P = 0.016	p<0.001
	*TGFB1*	0.9 (0.8–1.1)	0.8 (0.7–1.0)	NS	1.2 (0.9–1.4)	1.1 (1.0–1.3)	NS	ND
	*FOXP3*	1.2 (0.9–1.5)	1.5 (1.1–1.9)	P = 0.022	1.1 (0.8–1.7)	1.5 (1.2–1.8)	P = 0.048	P = 0.007

IFN-β-treated MS patients were sampled 36–48 hours after injection of IFN-β. Gene expression analysis was done by quantitative real-time PCR. Data were obtained in a discovery group (cross-sectional study) and a validation group (longitudinal study) for screening and reproduction of significant findings. Data were obtained from the sub-study 3 population and are given as median normalization ratio with interquartile range in parenthesis. Differences in gene expression were only considered to be significant, and tested for significance in pooled data, when they were unidirectional with a p-value< 0.05 in both studies. ND = not determined; NS = not significant.

Genes encoding the T_H_1 transcription factor T-bet and HLX-1, the T_H_1 effector cytokine IFN-γ and T_H_17 polarizing cytokines IL-23 and IL-1β, were expressed significantly lower in whole blood from IFN-β-treated than from untreated MS patients. Conversely, the expression of genes encoding the immuno-regulatory cytokines IL-27 and IL-10 and the T_REG_ transcription factor FoxP3 was increased in IFN-β-treated patients. Thus, assessed on bulk preparations of whole blood, the pattern of IFN-β treatment-induced changes in gene expression consistently pointed to an inhibition of pro-inflammatory T_H_1 and T_H_17 activity and strengthened immunoregulatory activity.

### IFN-β treatment effects on CD4^+^ T-cell and monocyte gene expression

To confirm our findings and interpretations on significant IFN-β treatment effects on the gene-expression in whole blood, we sought to identify the blood cell subsets in which IFN-β treatment did induce changes in the expression of the respective genes. For these studies we first screened mRNA-expression levels in whole blood, PBMCs, CD4^+^ and CD8^+^ T-cells, NK-cells, B-cells, dendritic cells and monocytes, isolated by immunomagnetic labeling and magnetic separation from blood samples from 4 untreated and 4 IFN-β-treated patients (sub-study 4 population). It turned out that most T_H_ cell signature molecules were broadly expressed and only at low levels in CD4^+^ T-cells (S1 Fig.). These studies, however, also indicated that monocytes appear to contribute substantially to the increased *IL10* and *IL27* gene expression levels, which we had found in whole blood of IFN-β-treated patients (S1 Fig.).

Therefore, we decided to examine further blood samples from untreated and IFN-β-treated MS patients to gain statistically robust data on the treatment effects of IFN-β on the gene expression in CD4+ T-cell and monocytes *in vivo*.

**Fig 2:**
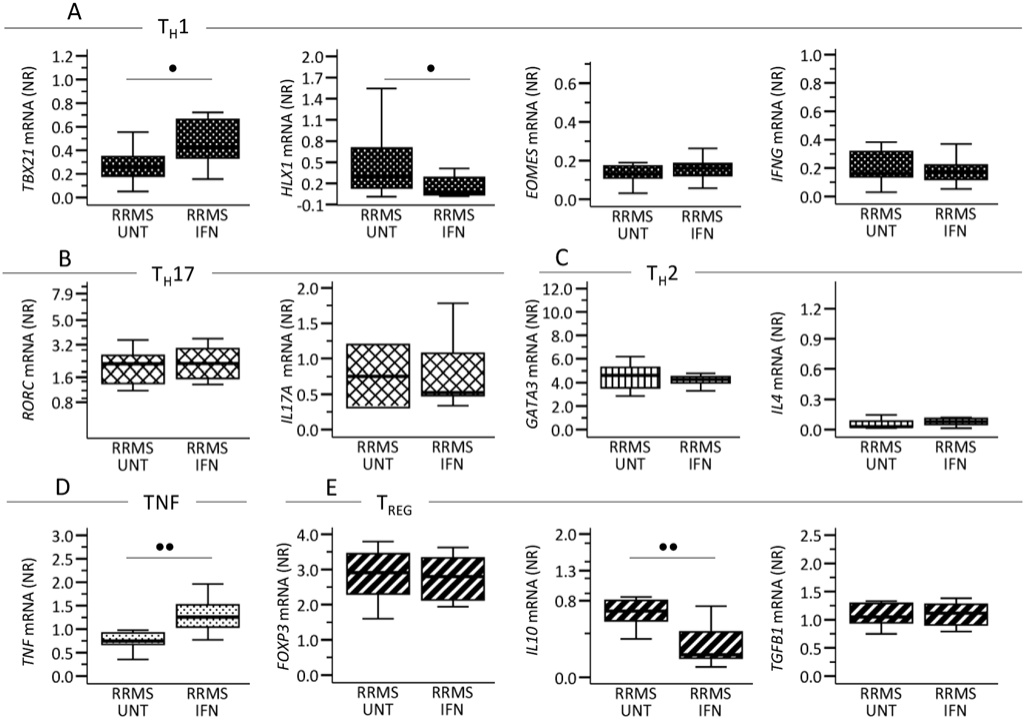
Circulating CD4^+^ T-cells’ *in vivo* mRNA expression of (A) T-helper (T_H_) 1, (B) T_H_17, (C) T_H_2, (D) *TNF* and (E) T-regulatory (T_REG_) associated molecules in 11 untreated (UNT) and 12 interferon(IFN)-β treated patients with relapsing-remitting multiple sclerosis (RRMS). IFN-β treated patients were sampled 36–48 hours after injection of IFN-β. CD4^+^ T-cells were isolated by immunomagnetic labeling and separation. Gene expression levels are given as normalization ratio (NR), boxes represent interquartile range, median value indicated as a line; whiskers represent range. Studies were made on the sub-study 2 population. Statistics was by the Mann-Whitney U test. • = p<0.05, •• = p<0.005.

In CD4^+^ T-cells, separated from blood samples from 11 untreated and 12 IFN-β-treated MS patients (sub-study 2 population), we measured mRNA-expression levels of: the T_H_1 markers *TBX1*, *HLX1*, *EOMES* and *IFNG* (Fig. 2A); the T_H_17 markers *RORC* and *IL17A* (Fig. 2B); the T_H_2 markers *GATA3* and *IL4* (Fig. 2C); *TNF* (Fig. 2D); and the T_REG_ markers *FOXP*3, *IL10* and *TGFB1* (Fig. 2E). Compared to CD4^+^ T-cells from untreated MS patients, CD4^+^ T-cells from IFN-β-treated patients expressed decreased levels of immunoregulatory *IL10* and T_H_1 TF *HLX1* and higher levels of T_H_1 TF *TBX21* and pro-inflammatory *TNF*.

**Fig 3:**
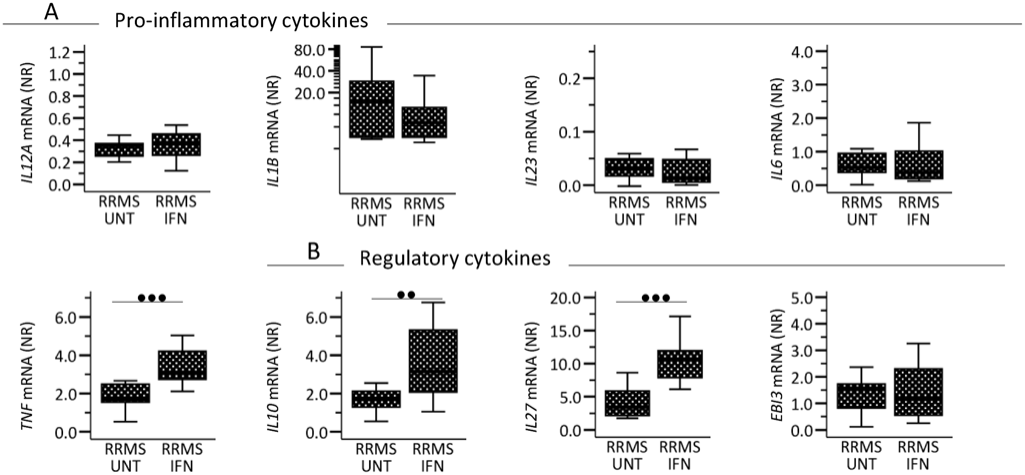
Circulating monocytes’ mRNA expression of genes encoding (A) pro- inflammatory and (B) immunoregulatory cytokines in 11 untreated (UNT) and 14 interferon-(IFN) β treated patients with relapsing-remitting multiple sclerosis (RRMS). IFN-β-treated patients were sampled 36–48 hours after their last injection of IFN-β. CD14^+^ cells were isolated by immunomagnetic labeling separation. Gene expression levels are given as normalization ratio (NR), boxes represent interquartile range, median value is indicated as a line, whiskers represent range. Studies were made on the sub-study 2 population. Statistics was by the Mann-Whitney U test. • = p<0.05, •• = p<0.005; ••• = p<0.001.

In monocytes, separated from blood samples from 11 untreated and 14 IFN-β treated MS patients (sub-study 2 population), we measured mRNA expression levels of the pro-inflammatory molecules *IL12A*, *IL1B*, *IL23*, *IL6* and *TNF* (Fig. 3A) and the immunoregulatory molecules *IL10*, *IL27* and *EBI3* (Fig. 3B). As suggested by our initial gene-expression studies, we found that expression of *IL10* and *IL27* in monocytes was significantly higher in IFN-β-treated than in untreated MS patients. Furthermore, IFN-β-treated patients displayed increased *TNF* expression by monocytes.

### Exogenous and endogenous IL-10 inhibits CD4^+^ T-cell reactivity to MBP *in vitro*

Increased mRNA expression of the immunoregulatory cytokines IL-10 and IL27 in monocytes was the most prominent and best reproducible treatment effect of IFN-β in our gene expression studies. Furthermore, increased IL-10 expression had also been associated with spontaneous expression of IFN-β-inducible genes and disease control in untreated MS [18]; and restriction of disease severity in EAE had been associated with type-I IFN signaling and the expression of IL-27 in macrophages [7]. Therefore, we decided to examine the extent to which monocytes, IL-10 or IL-27 may account for decreased reactivity of MBP-specific CD4^+^ T-cells (sub-study 5 population).

**Fig 4:**
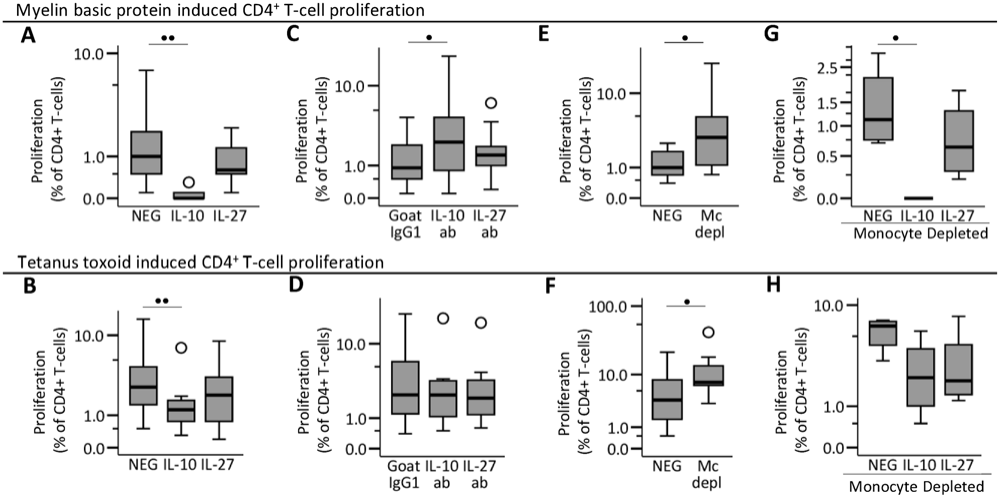
The effect of IL-10, IL-27 and monocytes on MBP-specific CD4^+^ T-cell activation. Freshly isolated PBMCs were stained with CFSE and cultured 7 days in RPMI-medium supplemented with 5% AB-serum and myelin specific protein (MBP;30 μg/ml) or tetanus-toxoid (TT;10 μg/ml). day 7 cells were stained with fluorochrome-conjugated antibodies and analyzed by flow cytometry. Effects on (A) MBP-induced and (B) TT-induced CD4^+^ T-cell proliferation when IL-10 (5ng/ml) or IL27 (40 ng/ml) was added to the culture (n = 9); effects on (C) MBP-induced and (D) TT-induced CD4^+^ T-cell proliferation when endogenous IL-10 or IL-27 was inhibited by adding neutralizing IL-10-antibody (6 μg/ml) or IL-27-antibody (6 μg/ml; n = 9) to the culture; effects of monocyte depletion (Mc depl) from the PBMC preparations on (E) MBP-induced and (F) TT-induced CD4^+^ T-cell proliferation (n = 7); effects on (G) MBP-induced and (H) TT-induced CD4^+^ T-cell proliferation when IL-10 or IL27 was added to monocyte-depleted PBMC cultures (n = 4). Boxes represent interquartile range, median value indicated as a line, whiskers represent range, ^₀^ = outliers, * = extremes. Studies were made on the sub-study 5 population. Statistical analysis was by paired t-test: • = p<0.05; •• = p<0.005.

In studies of MBP-induced and TT-induced CD4^+^ T-cell responses addition of IL-10 significantly decreased CD4^+^ T-cell proliferation (Fig. 4A and 4B). Blocking of endogenous IL-10 with anti-IL-10 antibodies significantly increased the MBP-induced CD4^+^ T-cell proliferation (Fig. 4C), whereas TT induced CD4^+^ T-cell proliferation was not affected significantly(Fig. 4D). At the limited number of conditions tested in our study, IL-27 had no significant effects on antigen induced T-cell proliferation in our assay (Fig. 4A-4C).

### Effect of IL-10 on MBP-reactive CD4^+^ T-cells in monocyte-depleted cultures

In cultures with monocyte-depleted PBMCs we found significantly increased MBP-induced and TT-induced CD4^+^ T-cell proliferation (Fig. 4E and Fig. 4F). The addition of IL-10 to cultures with monocyte-depleted PBMCs, significantly reduced MBP-induced CD4^+^ T-cell proliferation (Fig. 4G). Monocyte depletion also increased the frequency of proliferating CD4^+^ T-cells in response to TT (Fig. 4F), In contrast, TT-induced proliferation of CD4^+^ T-cells in monocyte-depleted PBMC cultures were not inhibited significantly by the addition of IL-10 (Fig. 4H).

## Discussion

In the present study we found that: 1) CD4^+^ T-cell activity to myelin basic protein (MBP) in untreated MS patients was associated with decreased expression of IFN-β-inducible genes in PBMCs; 2) IFN-β-treated MS patients had decreased CD4^+^ T-cell activity to MBP; 3) IFN-β-treated MS patients had increased expression of *IL10* and *IL27* in monocytes *in vivo*; and 4) monocytes had immunoregulatory effects, and exogenous and endogenous IL-10 inhibited antigen-specific CD4^+^ T-cells activation *in vitro*.

In agreement with our work, previous studies have reported reduced reactivity of myelin-specific CD4^+^ T-cells *ex vivo* in IFN-β treated MS [26,27]; these studies used an assay similar to our own with PBMCs cultured in AB-serum-supplemented media. However, another study, which used 30% autologous serum in the culture medium, did not find any decrease in MBP-induced CD4^+^ T-cell proliferation in IFN-β-treated MS [28]. Modulation of T-cell responses may represent one of various therapeutically relevant modes of action of IFN-β in MS [29]. In EAE type I IFNs restrict disease severity as well, but studies disagree on the role of systemic CD4^+^ T-cell activation for this phenomenon as some studies in IFN-β and type I IFN-receptor knockout mice suggest that effects within the CNS may be more important than systemic effects [7,11–14]. This does not exclude that type I IFNs may regulate CD4^+^ T-cell responses in humans. We found that genes, strongly induced in MS patients treated with IFN-β, are expressed at lower levels in untreated MS patients with increased CD4+ T-cell reactivity to MBP than in untreated MS patients without detectable reactivity to MBP. However, the present study does not examine the extent to which this may be associated with a particular disease course of the patients. The assumption that MS patients may benefit from spontaneously increased expression of IFN-β inducible genes is controversial. Two recent studies suggest that a subgroup of MS patients with high serum concentrations of IFN-β and IL-17F or with evidence of high expression levels of type I IFN-induced genes respond poorly to subsequent treatment with IFN-β [15,30]. This was, however, not confirmed by other studies [18,20,22,31]. Furthermore, high expression levels of IFN-β-inducible *MX1* were associated with lower disease activity in untreated MS patients [18,20].

The interpretation of the limited number of differentially expressed genes, the majority of them IFN-β-inducible, in MS patients with low TT-induced CD4+ T-cell proliferation is difficult. Data suggest that TT-reactivity also may be susceptible to IFN-β inducible genes but to a lower extent than MBP-reactivity. A general problem when using TT as control and recall antigen is, however, that the vaccination history of each individual has an important impact, which we could not take into account. This and different assays and readouts may also explain that the extent of TT-induced T-cell recall responses may vary in different studies.

The most conspicuous and best reproducible finding in our gene expression studies was increased *in vivo* expression of *IL10* and *IL27* in monocytes from IFN-β-treated MS patients. Previous studies have shown that IFN-β induces IL-10 production in monocytes *in vitro* [32,33], and numerous CSF and blood biomarker studies have linked high levels of *IL10* gene expression or IL-10 protein *in vivo* to IFN-β treatment and to disease control in IFN-β-treated and untreated MS [18,33–41]. Likewise, low serum levels of IL-27 have been associated with multiple sclerosis and high production of IL-27 by PBMCs has been associated with a less severe disease course in IFN-β-treated MS [9,42]. In a recent study we found that increased expression of the IFN-β-inducible *MX1* gene correlated with high expression of the *IL10* gene in blood cells, and *IL10* gene expression was lower in untreated MS patients with treatment-induced, neutralizing anti-IFN-β antibodies [18]. This suggests that genes, induced by endogenous IFN-β, may play a non-redundant role in regulating *IL10* expression in blood cells. Importantly, high expression of *MX1* was associated with lower disease activity in two independent studies [18,20]. Our cell sorting studies suggest that monocytes are one of the major cell types responsible for the increase in *IL10* and *IL27* expression *in vivo* in patients treated with IFN-β. However, the present study does not prove that this also applies to individuals who have increased expression of IFN-β-inducible genes.

In accordance with IL-10’s physiological role to regulate and resolve adoptive immune responses [43–45], endogenous and exogenous IL-10 restricted antigen-induced CD4^+^ T-cell responses in our assay. Monocyte depletion resulted—similar to IL-10 neutralization—in an enhanced CD4^+^ T-cell response to MBP, and this effect could be reversed by the addition of IL-10 to the cultures. The proliferative response to tetanus-toxoid (TT) also increased in monocyte depleted cultures, but this effect could not be reversed significantly by addition of IL-10 to the culture. Thus our results agree with other studies showing that monocyte-derived IL-10 may inhibit T-cell activation [45–47]. However, the inhibition of TT-induced T-cell responses by IL-10 seemed partly to depend on the presence of monocytes, demonstrating that IL-10 inhibits TT induced T-cell proliferation by monocyte-dependent effects, which is in agreement with a previous study [48]. Strikingly, IFN-β treated patients had increased expression of the *IL10* gene in circulating monocytes and reduced T-cell responses to MBP but not to TT despite the fact that L-10 was able to reduce TT induced CD4+ T-cell proliferation. A previous study showed that the autoantigen thyroglobulin induced a much stronger secretion of IL-10 from monocytes than the recall antigen TT in vitro [49]. What applies to the autoantigen thyroglobulin may also apply to the autoantigen MBP. In contrast to MBP, TT and TT-specific T-cells may fail to induce substantial secretion of IL-10 even though monocytes, previously exposed to IFN-β *in vivo*, may have a higher potential to produce IL-10. This could also explain why neutralizing IL-10 antibodies did not have a significant effect on TT-reactivity.

We also found increased expression of *IL27* in monocytes from MS patients treated with IFN-β. Previous *in vitro* studies suggest that APC-derived IL-27 inhibits T-cell activation and Th17 polarization [7–9]. In our system, assessing antigen specific T-cell responses, IL-27 did not affect T-cell responses substantially. However, we did not examine various conditions of IL-27 and IL-27 neutralization and therefor our study does not provide a complete characterization of the effect of IL-27 in our assay. There may also be several other reasons for these discrepancies: 1) autocrine IL-27 may modify APC secretion of other polarizing and regulatory cytokines involved in T_H_-cell activation [8,9]; 2) IL-27 may have different effects in rodents and humans or on antigen-specific and polyclonal CD4^+^ T-cell stimulation [7–9]; 3) there may be functional differences in dendritic cell and monocyte-derived IL-27 [8,9]. Thus, there is compelling evidence that IL-27 has a central role in the regulation of T-cell responses, and that IL-27 may acts synergistically with IL-10 [7]. A general limitation of our *in vitro* studies is that they examine the role of IL-10, IL-27 and monocytes in our T-cell assay to demonstrate that IFN-β potentially may interfere with T-cell responses by modulating IL-10, IL-27 and monocytes. We did not, however, examine how IFN-β and other type I IFNs actually modulated IL-10, IL-27 and monocytes and affected T-cells *in vitro*.

A longitudinal study design, assessing the disease course and T-cell activation of MS patients before and during treatment with IFN-β would have made it easier to correlate IFN-β-induced immune effects and examine the extent to which they correlate with the clinical response to treatment. IFN-β modifies the expression of myriads of genes in immune-cells which is a major challenge for studies trying to identify therapeutically relevant modes of action of IFN-β treatment in MS [4,29]. Likewise we found that expression levels of a wide range of molecules were affected in IFN-β-treated MS patients: 1) *IL10* expression was significantly decreased in circulating CD4+ T-cells, which however was not associated with an decreased potential of antigen-specific CD4+ T-cells to express IL-10 ex vivo. This highlights the fact that static *in vivo* measures do not necessarily reveal the potential of what cells are capable of doing; 2) TNF-α mRNA was significantly increased in circulating monocytes and CD4^+^ T-cells in IFN-β treated patients. TNF-α is mostly known for its pro-inflammatory properties, however, as anti-TNF treatment leads to exacerbation in MS and TNF-α also has immune-regulatory immune-effects the role of increased *TNF* in IFN-β treated MS may be complex [50]; 3) The transcription factors *TBX21 and HLX1* are primarily associated withT_H_1 cells, however *TBX21* also appears to have a role in T_H_17 differentiation [51,52]. In T-cells type-I IFNs induce STAT1 that is an inducer of *TBX21* [53,54], which may explain the increased *TBX21* expression levels we found in CD4^+^ T-cells from IFN-β treated MS patients. In that perspective the decreased expression level of *HLX1* in circulating CD4^+^ T-cells from IFN-β treated MS patients is difficult to explain and may be related to the wide range of molecules and pathways type-I IFNs interfere with.

In conclusion, the present study shows that increased expression of IFN-β-inducible genes in PBMCs from untreated MS patients and treatment with IFN-β is associated with reduced MBP-induced T-cell responses. We also found that IFN-β-treated MS patients have increased expression levels of *IL10* mRNA in circulating monocytes and that monocytes and IL-10 suppress MBP-specific T-cell responses *in vitro*. Previous studies have shown that high expression levels of *MX1* were associated with lower disease activity in MS patients and correlated with high expression levels of the *IL10* gene in blood cells [18,20]. Therefor we hypothesize that decreased MBP-induced T-cell responses in MS patients with increased endogenous expression of IFN-β inducible genes may be mediated by monocyte-derived IL-10 and may be associated with a milder disease course. This hypothesis does, however, need to be confirmed in future studies.

## Supporting Information

S1 Fig Expression of genes associated with regulation, differentiation and activation of T-helper (T_H_) and T-regulatory cells in whole blood, periphery blood mononuclear cells (PBMC) and in major PBMC-subsets: CD4^+^ T-cells (CD4Tc); CD8^+^ T-cells (CD8Tc); NK-cells (NK); B-cells (Bc); monocytes (Mc); dendritic cells (Dc). Samples were obtained from 4 untreated and 4 interferon-(IFN) β treated patients with relapsing remitting multiple sclerosis (RRMS; sub-study 4 population). IFN-β-treated patients were sampled 36–48 hours after their last injection of IFN-β; PBMC-subsets were isolated by immunomagnetic labeling and separation. Gene expression is given as normalization ration (NR), boxes represent inter-quartile range, median value indicated as a line; whiskers represent range. Due to low sample size, data given are descriptive and were not tested statistically. ¥ = gene expression was measurable in only 2 or less samples.
(PDF)

S1 Table Targets used in gene expression analysis by PCR. (PDF)

S2 Table Antibodies and other reagents used to stain cells for flow cytometry analysis. (PDF)
