# Supplementary figures and images for: Endogenous Interferon-β-Inducible Gene Expression and Interferon-β-Treatment Are Associated with Reduced T Cell Responses to Myelin Basic Protein in Multiple Sclerosis
Source: PLoS One. 2015 Mar 4;10(3):e0118830. doi: 10.1371/journal.pone.0118830 (PMC4349448; doi:10.1371/journal.pone.0118830)

**FIGURE S1**

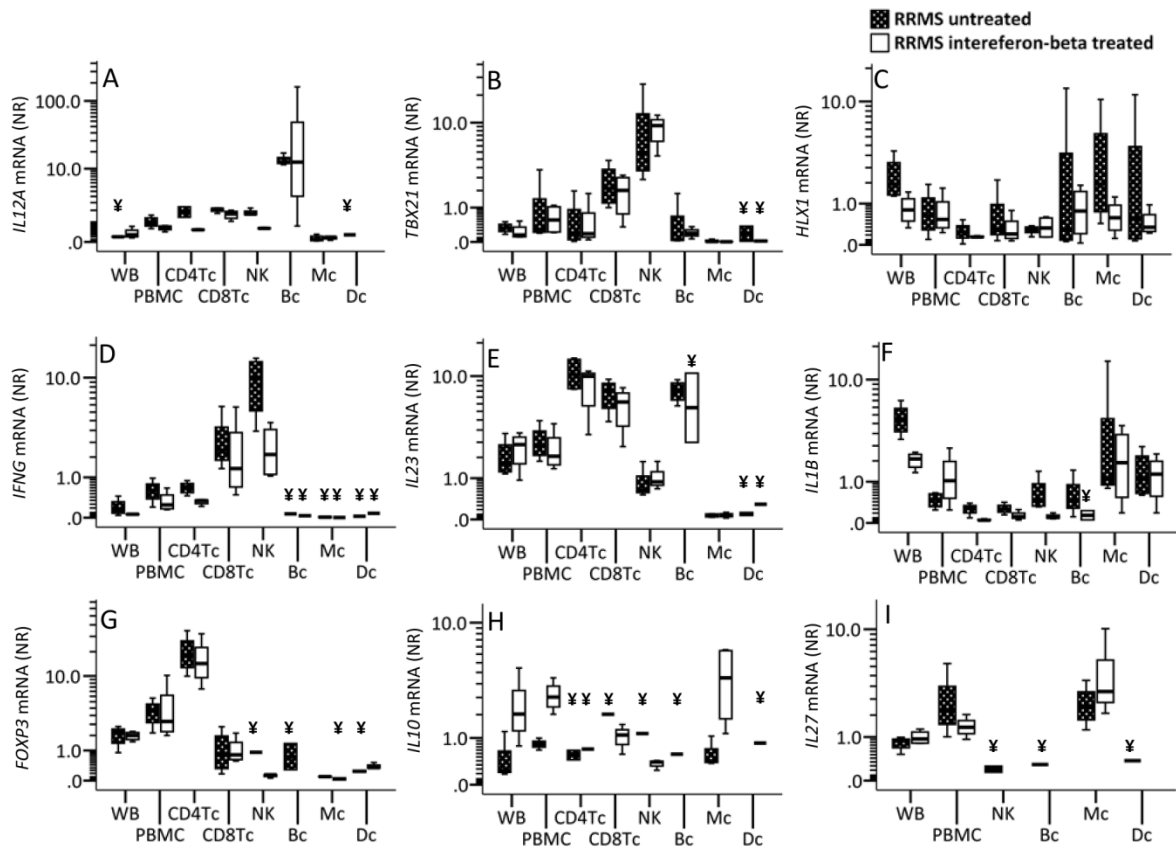

Supplement: S1 Fig — Samples were obtained from 4 untreated and 4 interferon-(IFN) β treated patients with relapsing remitting multiple sclerosis (RRMS; sub-study 4 population). IFN-β-treated patients were sampled 36–48 hours after their last injection of IFN-β; PBMC-subsets were isolated by immunomagnetic labeling and separation. Gene expression is given as normalization ration (NR), boxes represent inter-quartile range, median value indicated as a line; whiskers represent range. Due to low sample size, data given are descriptive and were not tested statistically. ¥ = gene expression was measurable in only 2 or less samples. (PDF) [file pone.0118830.s001.pdf]
