# Supplementary material for: Endogenous Interferon-β-Inducible Gene Expression and Interferon-β-Treatment Are Associated with Reduced T Cell Responses to Myelin Basic Protein in Multiple Sclerosis
Source: PLoS One. 2015 Mar 4;10(3):e0118830. doi: 10.1371/journal.pone.0118830 (PMC4349448; doi:10.1371/journal.pone.0118830)
Supplement: S1 Table — (PDF) [file pone.0118830.s002.pdf]

TABLE S2

Symbol	Number	PCR analysis of whole blood	Explorative PCR studies on sorted PBMC-subsets	PCR studies on blood derived CD4+ T-cells and monocytes
EBI3	Hs00194957_m1	Y		Y
EOMES	Hs00172872_m1			Y
FOXP3	Hs00203958_m1	Y	Y	Y
GAPDH	Hs99999905_m1	Y	Y	
GATA3	Hs00231122_m1	Y		Y
HLX1	Hs00172035_m1	Y	Y	Y
IFNG	Hs99999041_m1	Y	Y	Y
IL12A	Hs00168405_m1	Y	Y	Y
IL17A	Hs00174383_m1			Y
IL18	Hs00155517_m1	Y		
IL1B	Hs00174097_m1	Y	Y	Y
IL1RN	Hs00277299_m1	Y		
IL23	Hs00372324_m1	Y	Y	Y
IL27	Hs00377366_m1	Y	Y	Y
IL4	Hs00174122_m1	Y		Y
IL6	Hs00174131_m1	Y		Y
IL10	Hs00174086_m1	Y	Y	Y
MAF	Hs00193519_m1	Y		
CASC3	Hs00904832_m1			Y
RORA	Hs00536545_m1	Y		
RORC	Hs01076116_g1	Y		
RORC	Hs01076112_m1			Y
TBX21	Hs00203436_m1	Y	Y	Y
TGFB1	Hs99999918_m1	Y		Y
TNF	Hs00174128_m1	Y		Y
UBE2D2	Hs00366152_m1			Y
