# Supplementary material for: Endogenous Interferon-β-Inducible Gene Expression and Interferon-β-Treatment Are Associated with Reduced T Cell Responses to Myelin Basic Protein in Multiple Sclerosis
Source: PLoS One. 2015 Mar 4;10(3):e0118830. doi: 10.1371/journal.pone.0118830 (PMC4349448; doi:10.1371/journal.pone.0118830)
Supplement: S2 Table — (PDF) [file pone.0118830.s003.pdf]

TABLE S3

Specificity	Isotype	Clone	Host	Fluorochrom	Provider
CD3	IgG1, κ	UCHT1	Mouse	Pacific Blue	BD Biosciences, Denmark
CD4	IgG1, κ	RPA-T4	Mouse	PerCP/Cy5.5	Biolegend, USA
CD8	IgG1, κ	HIT8a	Mouse	PE-Cy7	BD Biosciences, Denmark
CD19	IgG1, κ	HIB19	Mouse	APC-Cy7	Biolegend, USA
IL-17	IgG1, κ	BL168	Mouse	PE	Biolegend, USA
IFN-γ	IgG1, κ	B27	Mouse	APC	Biolegend, USA
IL-4	IgG1, κ	8D4-8	Mouse	PE	BD Biosciences, Denmark
TNF-α	IgG1, κ	MAb11	Mouse	APC	BD Biosciences, Denmark
IL-13	IgG1, κ	JES10-5A2	Rat	PE	BD Biosciences, Denmark
IL-10	IgG2a, κ	JES3-19F1	Rat	APC	BD Biosciences, Denmark
	IgG1, κ	MOPC-21	Mouse	PE	Biolegend, USA
	IgG2b, κ	MPC-11	Mouse	PE	Biolegend, USA
	IgG1, κ	RTK2071	Rat	PE	Biolegend, USA
	IgG2a, κ	R35-95	Rat	APC	BD Biosciences, Denmark
	IgG1, κ	MOPC-21	Mouse	APC	Biolegend, USA
CellTrace™ CFSE Cell Proliferation Kit					Molecular Probes, Denmark
Live/Dead® Fixable Aqua Cell Stain Kit for 405 nm excitation					Invitrogen, Denmark
